# Virtual Reality for Gerontological Education: A Pilot Study of Interdisciplinary Healthcare Training

**DOI:** 10.1093/geroni/igaf122.3683

**Published:** 2025-12-31

**Authors:** Mingyang Zheng, Sarah Rakes

**Affiliations:** Radford University, Blacksburg, Virginia, United States; Radfford University, Radford, Virginia, United States

## Abstract

The United States is facing a workforce shortage in caring for its aging population. To address this issue, we developed a training program using Virtual Reality (VR) technology to help healthcare professionals and students better understand and care for older adults, with a specific focus on Alzheimer’s disease and social isolation. Participants included nurses, therapists, counselors, students, and other healthcare professionals. We conducted pre- and post-training assessments measuring Alzheimer’s knowledge (Carpenter et al., 2009), confidence in working with older adults (Elvish et al., 2018), awareness of social isolation risk factors and resources, and overall training satisfaction. The program trained 107 participants, and among the 48 who completed both pre- and post-training assessments, knowledge about Alzheimer’s disease increased significantly, from a mean score of 16.63 to 22.15 (t = 4.38, p < 0.001). Similarly, confidence in working with older adults improved from 25.38 to 31.29 (t = 4.671, p < 0.001). Participants also showed increased awareness of social isolation, identifying more risk factors (from 3.33 to 3.81) and available resources (from 3.06 to 3.56) after the training. Nearly all participants (42 out of 44) reported learning something new, and 39 said they could apply what they learned in their work. The vast majority (43 out of 44) felt the training surpassed their initial expectations. This pilot project demonstrates that an immersive, VR-based approach to geriatric education significantly improves both knowledge and confidence among healthcare professionals and students.

